# Exploring the use of a dementia game for raising public awareness in Singapore: a descriptive qualitative study

**DOI:** 10.1186/s12889-025-26166-7

**Published:** 2026-02-16

**Authors:** Elaine Kee Chen Siow, Stephanie Craig, Shawn Tan Hao Xuan, Peter Kay Chai Tay, Gary Mitchell, Christine Brown Wilson

**Affiliations:** 1 Singapore Institute of Technology, Health and Social Sciences, Singapore, Singapore; 2 School of Nursing and Midwifery, Queen’s University Belfast, Belfast, Northern Ireland

**Keywords:** Dementia, Gamification, Public awareness

## Abstract

**Background:**

As the population of individuals living with dementia is estimated to increase significantly, addressing stereotypes and stigma associated with the condition has become crucial. Despite the widespread negative cultural depictions contributing to this stigma, the research on evidence-based interventions remains limited. Drawing from successful dementia awareness initiatives in the UK, this study aims to explore the use of a dementia game to raise public attitudes and knowledge of dementia in Singapore.

**Methods:**

An interpretive qualitative approach was adopted using focus group discussions (FGs). Participants were recruited through advertisements and word-of-mouth from the Singapore Institute of Technology and Dementia Singapore. A total of 19 individuals played a Dementia awareness game followed by participation in the FGs. The FGs were conducted virtually and video-recorded to facilitate data analysis. Thematic analysis was used to reveal patterns and themes arising from the data.

**Results:**

The results showed self-reported positive shifts in attitudes and knowledge. The majority of participants reported increased understanding and empathy towards persons living with dementia. Participants described gaining new knowledge about dementia, challenging stereotypes, and clarifying misconceptions. Participants who did not experience significant changes had previously worked in dementia care with prior training. Participants also provided valuable feedback on the game’s potential as an educational tool, suggesting improvements such as incorporating storytelling elements and identifying future target audiences in children.

**Conclusion:**

The dementia game enhances understanding, fosters empathy and dispels misconceptions, suggesting potential for diverse educational applications and the need for further research on this topic.

**Supplementary Information:**

The online version contains supplementary material available at 10.1186/s12889-025-26166-7.

## Background

The number of people living with dementia is expected to increase from 57.4 million cases in 2019 to 152·8 million cases in 2050 [1]. This growth in the number of persons living with dementia highlights the need for effective public health planning and policies to address the needs of the dementia population. In Singapore, one in ten individuals aged 60 and above is diagnosed with dementia [2].

Caring for people living with dementia requires a strong community effort and commitment. Social factors, such as social engagement, interpersonal connections, and social networks, play an important role in the well-being of people living with dementia [3], highlighting the importance of creating awareness and improving public perception to garner positive support from communities. However, in Singapore, stigma and misconceptions about dementia remain key challenges. Earlier national surveys, such as the 2019 Singapore Management University (SMU) study [4], revealed that many people living with dementia and their caregivers experienced rejection, loneliness, shame, and incompetence. More recent research indicates gradual improvements but persisting gaps in public understanding. Siddiqui et al. [5] found that lower socioeconomic status, limited personal exposure to dementia, and poorer baseline knowledge were associated with less favourable attitudes among middle-aged residents. Hansra et al. [6] reported that public attitudes toward dementia have improved compared to 2012, with significant gains in empathy and reductions in stigma-related responses, although misconceptions persist among individuals without direct contact with dementia. Findings from the 2023 nationwide Remember.For.Me. survey by Dementia Singapore and SMU [7] further supported these trends over 80% of respondents agreed that persons with dementia can lead fulfilling lives, yet many acknowledged uncertainty about how to support them. In healthcare contexts, Han et al. [8] also observed that even trained professionals in Singaporean community hospitals demonstrated variable knowledge and attitudes toward dementia. Together, these findings suggest that while public awareness and attitudes have improved in recent years, continued education and innovative awareness initiatives remain essential to address enduring misconceptions and stigma.

Initiatives like the “Forget Us Not” campaign play an important role in the creation of dementia-friendly communities in Singapore [9]. However, further steps are needed to ensure people living with dementia receive the respect and support necessary for active and purposeful living within the community. A study on perceptions towards dementia showed that false beliefs and attitudes were often linked to inadequate knowledge and limited personal experiences with the condition [10]. Stereotypes and depiction of dementia in the local culture are also often linked with negative thoughts and feelings, leading to a stigma of the condition which is often felt by those with dementia [11]. There is still insufficient evidence-based research on methods to combat the stigma associated with the condition [12], suggesting that additional efforts are required to improve the public perception of dementia in Singapore.

Globally, a variety of awareness-raising interventions have been implemented to improve public understanding and reduce stigma toward people living with dementia. These include public education campaigns, community-based workshops, simulation activities, and interactive learning programmes such as Dementia Friends in the UK and the ‘Forget Us Not’ initiative in Singapore. Evaluations of such initiatives have shown that educational and participatory activities can increase empathy, improve knowledge, and promote more inclusive community attitudes toward dementia. For instance, structured awareness programmes and social contact interventions have demonstrated measurable improvements in dementia knowledge and reductions in negative stereotypes [13, 14]. However, traditional approaches often rely on didactic teaching methods and may not effectively engage younger or digitally oriented audiences. Consequently, serious games and gamified interventions have emerged as a novel means of raising awareness in an interactive and engaging format, with growing evidence supporting their potential to enhance learning outcomes and attitude change [15].

Serious games are intended for an educational purpose but are designed to be fun and engaging [16]. In recent years, serious games have been increasingly used to educate and train healthcare professionals and the public about a range of healthcare topics including dementia [17]. For example a Dementia Awareness Game was found to improve attitudes of the general public [18] and knowledge and attitudes in nursing students [19]. Another digital game on influenza also proved successful in improving knowledge and intentions among nursing students to become vaccinated [20]. Hence, this suggests that the use of serious games may challenge people’s thinking and improve knowledge and perceptions.

## Introduction

**Fig. 1:**
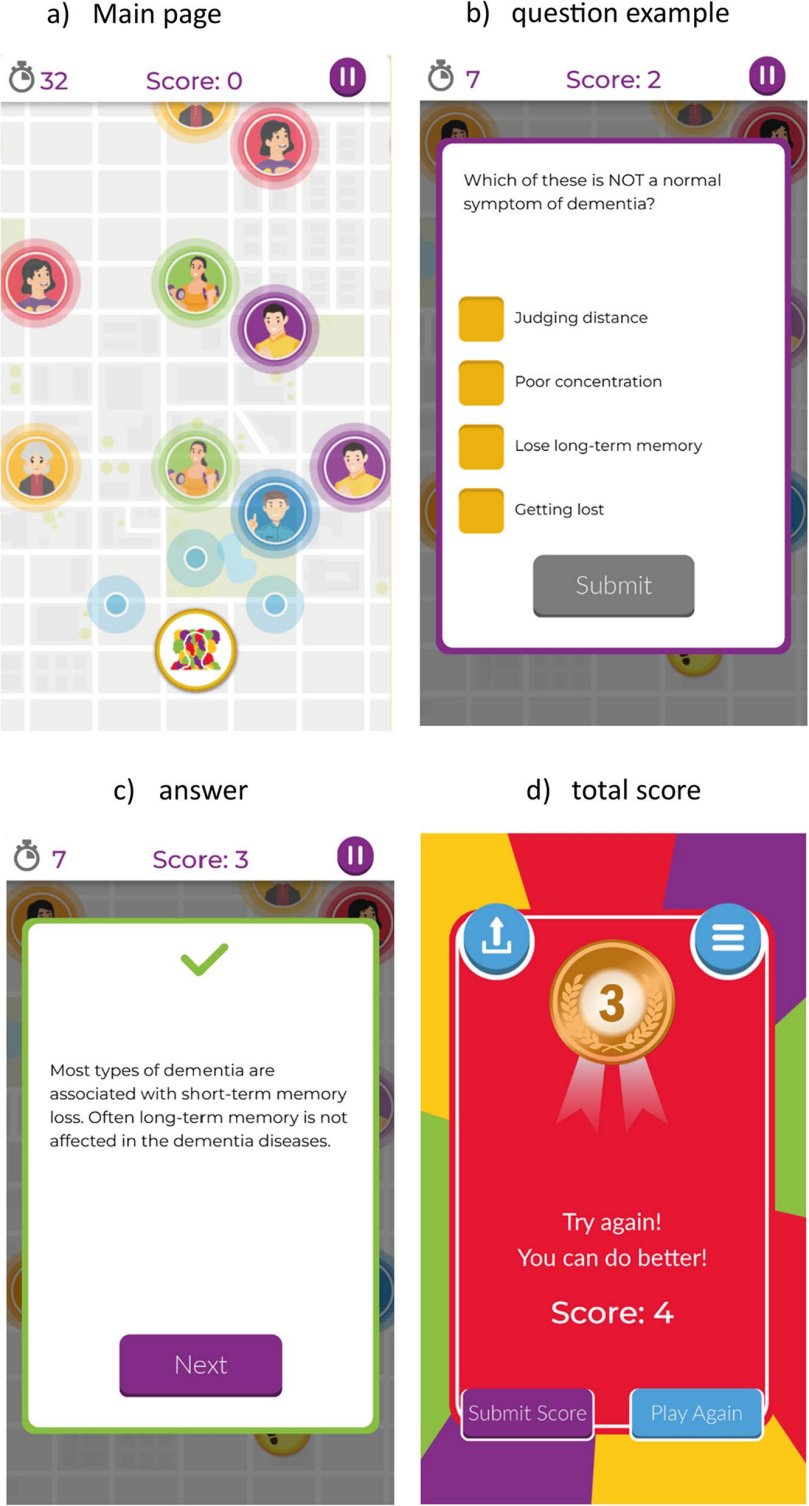
Example screens of dementia awareness game during play. Example screens of dementia awareness game during play (with kind permission from Focus Games(TM) accessible from www.dementiagame.com. : **a**)main page, **b**) question example, **c**) answer, **d**) total score

The Dementia Game (www.dementiagame.com) was co-designed by three of the authors (CBW, GM, SC) with people living with dementia to improve public attitudes toward dementia. This should read, People living with dementia from a local empowerment group, Dementia Northern Ireland (NI), undertook focus groups that identified the themes of the game within the codesign process [21]. The game (Fig. 1) was assessed using a pre/post-test using Approaches to Dementia Questionnaire (ADQ) [22]. More than 1000 individuals from Northern Ireland played over 4 weeks with 500 people completing the pre/post questionnaire. Findings from this study demonstrated that after playing the game, there was a statistically significant improvement across all domains of the questionnaire, showing more optimistic perceptions of the abilities and the future of people living with dementia, recognising them as unique individuals with the same value as anyone else, and overall demonstrating a more positive attitude towards people living with dementia [18]. The dementia game was also used with a cohort of 334 year-one nursing students in Queen’s University Belfast (QUB), Northern Ireland who completed pre/post-tests and the total ADQ score again demonstrated a significant improvement in positive attitudes, with both subscales of Hope and Recognition of Personhood seeing significant improvements [19].

Although the benefits of using serious games to improve perceptions of dementia have been demonstrated in the UK, further research is required to understand the effects in an ethnically diverse population [18]. Hence, the purpose of this project is to evaluate if playing this dementia awareness game can improve the general public’s attitudes and knowledge of dementia in Singapore.

The Dementia Game is a web-based educational game designed to improve public understanding and attitudes toward dementia through interactive learning. Players progress through a virtual board by answering multiple-choice questions about dementia, covering topics such as symptoms, risk factors, daily living, and myths or misconceptions. Immediate feedback is provided after each response, with short explanations reinforcing accurate information and challenging stereotypes. The game can be completed in approximately 10–15 min and can be replayed for reinforcement (Fig. 1).

## Methods

The aim of this study was to explore if the Dementia Game might improve the general public’s perception, knowledge and attitudes towards people living with dementia in Singapore. The specific aims are to (i) explore the use of the dementia game as an educational tool for the general public in Singapore and (ii) identify areas for improvement to maximise its impact on dementia awareness in Singapore.

The version of the Dementia Game used in this study was the same as that evaluated by Carter et al. [18]. No cultural or linguistic adaptations were made, as the study aimed to examine the transferability of a UK-developed dementia awareness game to the Singapore context.

Therefore a descriptive qualitative study using focus groups following participants’ engagement with the Dementia Game was chosen. As this study is not a clinical trial, it has not been formally registered. This qualitative study was conducted sequentially following a separate quantitative evaluation of the Dementia Game [13]. The quantitative phase measured changes in knowledge and attitudes using the Approaches to Dementia Questionnaire (ADQ), while this qualitative phase explored participants’ experiences and perceptions in depth to understand how the game influenced awareness, empathy, and cultural relevance. Both studies were analysed independently to maintain methodological rigour and avoid data conflation. The qualitative approach was therefore designed to complement, rather than triangulate with, the quantitative findings by providing rich contextual insights into the subjective experiences of participants. This study followed the Consolidated Criteria for Reporting Qualitative Research (COREQ) 32-item checklist [23] to ensure comprehensive reporting of the study design, data collection, and analysis.

### Participants

Participants were recruited by two collaborators who were independent from this study. Information was distributed to an autonomous university in Singapore to students and faculty via email, and Dementia SG aided in reaching caregivers and the public. Advertising was expanded to social media and Telegram channels for wider outreach. The inclusion criteria for participants are outlined in Table 1. A $40 e-voucher was given to those who signed up and completed the FG as a token of appreciation for their time.

**Table 1:** Inclusion criteria

Inclusion Criteria
Participants must have access to an electronic device to participate in a Zoom meeting
Participants must be 18 years and older
Participants must complete and submit written informed consent before the start of the FG

Participants for the FG were recruited through convenience sampling [24] from an earlier quantitative study [13] where participants played the game and completed a pre/post-test survey (ADQ). The survey included an option for participants to leave their emails if they would like to participate in the FG. Of the 138 participants who completed the quantitative study, 64 had expressed an interest in the FG. An email invitation was then sent to these 64 individuals, to provide more information about the FG. 40 individuals agreed to participate and provided written consent. Email communication was used to finalise arrangements, ensuring each of the five groups consisted of 6–8 participants.

### Data collection

The Dementia Game and all focus group interviews were conducted in English. English was selected as it is one of Singapore’s official languages and the primary language of instruction in higher education and research. All participants were fluent in English, and this ensured consistency between the game content and focus group interviews.

Focus group interviews took place throughout November 2024. The duration of focus group interviews was between 45 and 60 min and took place via Zoom, an online platform. All focus group interviews were moderated by two facilitators (SKCE and PKCT). Participants were asked structured open-ended and probing questions (Supplementary File 1) regarding the game’s impact on attitudes, knowledge, and perceptions of people living with dementia. Additional inquiries delved into game experience and suggestions for enhancement and future research.

Participants played the Dementia Game approximately one to four months prior to the focus group discussions, depending on recruitment timing and participant availability. After reviewing consent details at the beginning of the focus group interviews, this was followed by a brief 6-minute Kahoot quiz featuring questions extracted from the dementia game, serving as a recap of their gameplay, before initiating the discussion. Participants reported that the recap activity was helpful in refreshing their memory of the game and facilitating engagement during the discussion.

### Analysis

Data were transcribed verbatim and analysed using Quirkos, a qualitative data analysis software. Using a constructivist approach, deductive thematic analysis was conducted to identify patterns and themes within the data Braun and Clarke’s [25] six-stage framework: familiarisation with data, generating initial codes, searching for themes, reviewing themes, defining themes, and producing the report. After reviewing the interview data, multiple coding rounds identified meaningful codes. Segments were coded and reviewed to ensure coherence. Themes were refined based on patterns, leading to a narrative description of findings. The data were cross-checked and reviewed by the research team *f*or accuracy. Data coding and initial theme development were conducted independently by two authors (STHX and SKCE). Coding discrepancies were discussed and resolved through consensus among the wider research team to ensure consistency and trustworthiness of analysis.

Thematic saturation was monitored throughout data collection and analysis. After each focus group, the researchers (STHX and SKCE) met to review preliminary codes and identify emerging patterns. By the fifth focus group, no new codes or concepts were identified, suggesting that thematic saturation had been achieved. This determination aligned with Braun and Clarke’s [25] reflexive approach, emphasising richness and depth of data within each theme rather than a fixed numerical threshold.

#### Trustworthiness

To ensure the trustworthiness of research data the four criteria of credibility, transferability, dependability, and confirmability were followed [25]. SKCE and PKCT were present in all sessions to ensure consistency and quality of data collection. At the end of each session, there was a debriefing session between the research team to provide feedback and consolidate learning points for improvement prior to the next FG. Verbatim quotes from the participants are included, providing a rich and detailed description of their experiences and contributing to the authenticity of the research outcomes. The research team collectively reviewed the raw data, field notes, and thematic analysis results, aiming to reach a common consensus and enhance the reliability of the study’s conclusions.

#### Ethics

The study obtained ethical approval from Singapore Institute of Technology Institutional Review Board (Project No. 2023063). Participants were informed prior to consent taking that they could withdraw from the study at any point in time, they can inform the interviewers either during the interview or after the interview if they do not wish to proceed with the interview and would like their data removed from the study. The participants’ names and emails were kept confidential stored separately from the transcripts on a password protected university server. For privacy, participants’ names have been replaced with unique identifiers and any identifying details have been removed from the transcripts. All methods were performed in accordance with the Declaration of Helsinki [26].

## Results

A total of 40 participants were recruited. However, 2 voluntarily withdrew due to unavailability and 19 accepted the invitation but did not attend the FG. A total of 19 participants attended and completed the FG, which were conducted across five focus groups with 6–8 participants in each group. Table 2 presents the demographic characteristics of the 19 participants.

**Table 2:** Demographic characteristics of participants

Gender		
	Female	10 (52.6%)
	Male	9 (47.4%)
Age		
	18–24	3 (15.8%)
	25–34	7 (36.8%)
	35–44	6 (31.6%)
	45–54	2 (10.5%)
	55–64	1 (5.3%)
Ethnicity		
	Chinese	15 (78.9%)
	Malay	1 (5.3%)
	Indian	3 (15.8%)
Have a family or close friend living with dementia?		
	Yes	10 (52.6%)
Work with people living with dementia?		
	Yes	2 (10.5%)
I have previously undertaken dementia training		
	Yes	2 (10.5%)

Three themes were identified that showcased the participants’ perspectives and attitudes towards people living with dementia. These were: ‘Influencing attitudes and knowledge’, ‘User experience and recommendations’ and ‘Intergenerational dementia education.

### Theme one: influencing attitudes and knowledge

Post-gameplay, participants reported improved knowledge toward dementia, describing empathy and understanding for people living with dementia compared to before they played the game. They also gained new factual insights about the condition, emphasising the game’s role as an educational tool fostering positive shifts in perceptions. Some participants mentioned previously being cautious of interacting with people living with dementia as they were afraid of getting harmed, but now feel confident enough to offer help after playing the game:

*“ I think in the past I will just rely on other people to step forward and help them. But if I encounter them now*,* I will be the one who step forward to assist them. Because I know that they wouldn’t do anything dangerous to me*,* they wouldn’t hurt me so I won’t be so scared to interact with them.”* (Group 5, participant 3, female).

Some participants realised that people living with dementia are more capable than commonly portrayed and that some can live independently. This experience led to a shift in their understanding and attitudes toward dementia.

*“ I think playing the game helped me realise and break out of some stereotypes. I think that they are more able than is often portrayed. And it helped me realise that it’s possible in general to live quite a normal life. I realise there are elderly people with mild dementia who live on their own. So that was quite eye-opening*.” (Group 5, participant 2, female).

Some shared about how the game provided insights into the lifespan of people living with dementia and details that were previously unknown to them.

“ *I think it has definitely changed for me like I shared before. In terms of number of years they live*,* details shared. That was quite shocking for me. So I think there’s a lot of things which we learned in the game*.” (Group 2, Participant 3, male).

Others gained new perspectives about the relationship between old age and dementia. There was a general assumption that ageing led to dementia, however, the game changed their beliefs with new information.

*“ So prior to the game*,* I was unaware that old age is not directly correlated with dementia. So that’s an interesting fact for me because I would assume*,* as you get older*,* your brain starts to deteriorate a bit*,* which might also cause dementia. But that apparently is not the case*,* I think that is something very interesting for me.*” (Group 3, Participant 3, male).

Similarly, the dementia game expanded the participants’ understanding by clarifying misconceptions and disseminating new information, such as revealing lesser-known risk factors like HIV infection, showcasing the game’s educational value in broadening the understanding of dementia’s complexities.

*“I think the game was a little bit more helpful in some of the underlying root causes of dementia like for instance HIV infection*,* I didn’t know about that so that was useful in helping to broaden my understanding with regards to dementia.*” (Group 1, Participant 3, female).

*“How the game was*,* it sorts of made it a bit more fun to learn some facts and clear some misconceptions. Things like HIV infection*,* things like that. I also wasn’t aware.”* (Group 2, Participant 1, female).

Participants gained clarity on various aspects of dementia, including the risk factors and its distinction from Alzheimer’s disease.

*“ I think my perception of them is clearer after the game because I learned new things about what are some of the risk factors and what are not. I think*,* having a great appreciation of what dementia really is. And it’s not synonymous with Alzheimer’s. I think that helps.”* (Group 5, Participant 1, female).

For participants who did not have prior knowledge or experience with dementia, the dementia game served as an educational tool providing new knowledge to help them fill gaps in their understanding of the condition.

*“ The game helped me enhance some of the knowledge that I don’t have at all for dementia because I don’t have first-hand experience working with dementia*,* so I think anything there is new information for me.”* (Group 2, Participant 2, female).

The game not only improved participants’ knowledge and skills in dementia care during caregiving scenarios but also fostered empathy and understanding towards these individuals. Participants also gained insights into dementia symptoms and learned how to better interact with those affected.

*“The game definitely helped with more nuggets of knowledge of dementia I guess? Because from my understanding*,* dementia is usually touch and go kind of knowledge*,* very brief details*,* for example not interacting with the outside world*,* lack of social life*,* but in terms of how to manage or interact*,* that’s the part that I find myself lacking in and after the game*,* I could sort of get a few clues how to better interact and manage dementia and people with dementia.”* (Group 1, Participant 1, male).

*“ Probably in terms of showing more empathy and understanding when interacting with someone with that condition. and probably because I’m more aware of what that condition entails and what are the possible different symptoms that they can show*,* so just in terms of being more patient once I’m aware they are suffering from this.*” (Group 2, Participant 1, female).

However, not all participants indicated that their attitudes and knowledge about dementia changed after interacting with the game.

*“For the questions I saw*,* I know some of the answers because I have training with people with dementia*,* we have done our own research*,* so this game did not really change my perception as I already have some knowledge and my research.*” (Group 1, Participant 4, male).

Upon further analysis, it became evident that these individuals had experience working with people living with dementia and had received prior training, which may have covered similar material within the game:

*“Because I already understand*,* like I work with people living with dementia*,* so I know what it’s like for them*,* so my perception didn’t really change.”* (Group 1, Participant 2, female).

Some indicated doubt regarding the game’s ability to change perceptions of dementia, citing prior experiences observing dementia-related decline, suggesting that these experiences may overshadow any potential influence from the game.

*“Whether the game changed my perception towards people with dementia or not*,* I don’t think it will make much difference. I already have seen people with dementia. How they deteriorate over time.”* (Group 4, Participant 3, female).

## Theme 2 - user experience and recommendations

Participants offered feedback to enhance the game, suggesting improvements in content, accessibility, and educational value. Ideas included adding interactivity and adjusting difficulty levels. Some raised concerns about knowledge retention and proposed alternative learning methods.

*“Sometimes if you play a game and then after a long time*,* ask about it*,* you may not remember. So*,* the retention rate is not very high*,* so maybe there can be other methods that can consistently build*…” (Group 2, Participant 5, female).

*“I did learn something during the game*,* but that was just a few minutes. I left it in my mind*,* then after that*,* I forgot about it. So*,* I don’t think the game is necessarily a good way to educate people about dementia. In addition*,* the game is frankly too short*,* and I didn’t really learn too much from it.”* (Group 4, Participant 1, male).

Others commented on the short durations and limited levels of the game for sufficient learning and gave suggestions to implement more stages and levels of questions to further engage the players.

*“I remember trying to play the game the first time*,* it was quite short to complete the stage. I think this one currently only has 1 level*,* once you walk to the end*,* that’s done already. You can redo*,* but the questions are the same. One suggestion is to have different tiers*,* levels*,* so it’s like the 1st level is the easier questions*,* and then it gets more difficult after a few levels.”* (Group 1, Participant 1, male).

Participants highlighted the importance of continuous engagement, and by addition of features such as multiple levels or a scoring system, can incentivise participation beyond a one-time experience.

*“If you want it to be more than that*,* even more engaging*,* like consistent. Then maybe you can try to add more features like more levels or something*,* or maybe can let players with high scores get a small token or something like that*.” (Group 5, Participant 3, female).

Participants suggested integrating the game at face-to-face community events like roadshows, offering a multi-sensory learning experience for direct interaction with caregivers and dementia patients. This engagement could amplify understanding and involvement, thus optimising the game’s impact.

*“Like roadshows those places*,* for example*,* Toa Payoh* [a centrally located residential town in Singapore] *hub or booths there. So maybe you can invite people to play there*,* cause I’m sure that they will be inclined to play. And also usually people of age will be like caregivers*,* so maybe they can let their kids*,* or whoever play the game*,* and they get to learn things like new knowledge.”* (Group 3, Participant 2, female).

*“I think if you really want to incorporate a game to educate people about dementia*,* it’d be better in a face-to-face setting*,* like roadshows. HDB* [Housing and Development Board] *Hub can organise a roadshow around the heartlands and then set up exhibition then you’re able to talk firsthand to caregivers and interact with dementia patients and then the game is incorporated into the roadshow. I think people will be more inclined to learn and it will give a more lasting impression. It’s a whole multi-sensory experience. Like you get to see the patient for herself. You get to talk to the caregivers. You understand what’s the experience like first-hand.”* (Group 4, Participant 1, male).

Participants also speak on the potential of storytelling in enhancing the educational value and engagement of the game. They suggest incorporating narrative elements, such as a fictional character going through relatable scenarios to people living with dementia, to create a more immersive learning experience.

“*I think on the retention bit*,* people connect well with stories like in this focus group*,* what people have shared from personal experiences*,* I think that will stay with me for a long time*,* and in a way also influence my perception moving forward. So with a game*,* if you can somehow incorporate a story*,* like a fictitious person*,* and you make that persona grow as people get the answers right*,* then that might make it more real because there looks like there’s a person attached to it as opposed to information*,* because I think some people may not have anyone in their life with dementia. So for them*,* it’s all concepts. It’s not real to a person in a way.”* (Group 2, Participant 6, female).

Incorporating information into these narratives can educate while also captivating the player emotionally, making facts more profound and memorable.

*“I mean*,* narrative is always powerful*,* right? So if it’s targeted at caregivers*,* maybe the main character could be somebody who is a caregiver learning about dementia.*

*So you’re kind of accompanying the character*,* right? To learn more*,* you could present different scenarios along the way*,* and then add in small snippets of facts like*,*. Yeah. a little bit like role-playing.”* (Group 5, Participant 2, female).

### Theme 3 – intergenerational dementia education

Participants highlighted the game’s potential to help children understand the behaviours of their loved ones with dementia, while also addressing misconceptions and stigma surrounding the condition. They described how a dementia game would be valuable for children to learn about dementia in a relatable and enjoyable way, fostering empathy and understanding.

*“Let’s say a primary school kid were to try to understand dementia a little bit more*,* maybe because their grandparents have dementia*,* then I think it’s a very easy way for them to gain a stronger understanding of why their loved one is acting a certain way.” (Group 1*,* Participant 3*,* female).*

*“I think it will be better for kids to play*,* it’s interactive. And they get to learn more about dementia*,* especially with Singapore’s ageing population. So it’s not a boring topic for them cause they get to play and learn at the same time.” (Group 3*,* Participant 2*,* female).*

Participants described how they thought that serious games were suitable for students and young people. By incorporating gamification elements, such as interactive gameplay, the game can effectively capture the interest and investment of younger audiences.

*“Because this is a web browser game*,* I would think that the younger generation are more into these kinds of things. By enabling gamification*,* I think this helps us to be more invested in whatever is being presented. So target demographic*,* younger people*.” (Group 3, Participant 3, male).

## Discussion

Increasing public awareness about dementia may help reduce the stigma, discrimination, and prejudice often faced by individuals living with the condition ( [27, 28]). This study aimed to explore if the Dementia Game might improve the general public’s perception, knowledge and attitudes towards people living with dementia in Singapore. Although the ‘Dementia Game’ has been evaluated in previous research [18], this was the first investigation into its impact in Singapore. A qualitative approach was used to explore how the dementia game developed in the UK might transfer to a culturally diverse English speaking high income country. The participants in this study provided detailed accounts as to how the Dementia Game enhanced their knowledge about dementia, fostering empathy and understanding, dispelling misconceptions, and highlighting the complexities of dementia. However, those with prior dementia experience saw little change in their perspectives, which warrants further exploration. The Dementia Game was codesigned for the general public in the UK to dispel common myths about dementia and to identify that people living with dementia retain capability to live independent lives in the community [18, 21].

Participants suggested improvements for the game, including increased interactivity, multiple difficulty levels, and storytelling elements, to boost engagement and knowledge retention. They also identified the game’s potential to educate children and young people, emphasising its role in addressing misconceptions and reducing stigma, thus making it a valuable intergenerational educational resource. This suggestion reflects participants’ recognition of the value of intergenerational education. Interestingly, a similar approach has been implemented in Northern Ireland through the development of the *Kids Dementia Game* (https://kids.dementiagame.com) [29], which was co-designed with children and people living with dementia to promote empathy and understanding from an early age. The inclusion of this example illustrates how the participants’ feedback in the Singapore context aligns with international developments and supports the potential for future adaptation of the Dementia Game for younger audiences.

Whilst the Dementia Game has been found to improve attitudes by increasing optimism and encouraging people to view those living with dementia as individuals [18, 19], this study further identified that gameplay enhanced participants’ empathy and confidence when interacting with people living with dementia. Participants without prior dementia-related experience described positive changes in understanding and behaviour, reporting a greater willingness to engage with and support people with dementia. In contrast, participants with previous training or caregiving experience reported minimal changes, suggesting that they were already familiar with many of the concepts presented in the game. These findings correspond with existing evidence that prior knowledge and direct contact with people living with dementia are associated with more positive and empathetic attitudes [30, 31].

This study also explains how the Dementia Game challenges stereotypes and common misconceptions about dementia. Participants explained how the questions in the game had supported them in developing a new perception of the capabilities and quality of life that people living with dementia can possess. Some were surprised to learn that some people living with dementia could lead relatively independent lives, which differed from conventional portrayal of dementia as a period of decline. As the dementia awareness game had been developed with the view of showcasing people living with dementia’s capability [21], this is a positive finding from this study.

The game provided insights into lifespans, risk factors, and different types of dementia, prompting players to re-evaluate their existing knowledge. Additionally, participants reported gaining practical and theoretical knowledge for dementia care, showcasing the game’s educational value. Prior studies on the dementia game that used the Approaches to Dementia Questionnaire (ADQ) reported similar results. Carter et al. [18] found that participants became more optimistic about the abilities and capabilities of People living with dementia after playing, while Craig et al. [19] showed significant improvement in knowledge of nursing students across various aspects of the condition, notably, in trajectory and risk factors of dementia. The current study provides further information findings of the previous two studies correlate with the present findings in this study, showing the game can have a positive effect in improving knowledge and attitudes in players.

Despite the majority experiencing positive changes, a minority of participants expressed that their attitudes and knowledge remained unchanged after game interaction. Upon further exploration, these individuals had professional experience in dementia care and did not feel the game add anything further to their knowledge. This finding aligns with existing evidence that direct or personal contact with stigmatised groups, including people living with dementia, is among the most effective means of reducing stigma and fostering positive attitudes. Participants with existing contact may therefore have experienced a ceiling effect, limiting further attitudinal change after gameplay. Future studies should examine the impact of the Dementia Game among individuals with little or no prior contact with people living with dementia to better evaluate its role as a public awareness intervention. While this observation suggests that healthcare professionals may require more advanced or context-specific educational materials, it should be interpreted cautiously given the small number of participants involved and the qualitative nature of this study. Carter et al. [18] similarly found statistically significant improvements among participants with prior dementia experience in the UK, based on a substantially larger sample (*n* = 171–253), which may have influenced the effect size observed. Further exploration is needed to understand how prior experience, professional training, and cultural context interact to influence the educational impact of dementia awareness games.

Participants in this study also provided constructive feedback and suggestions to improve the Dementia Game’s content and delivery. They proposed increasing levels of difficulty, integrating storytelling elements, and adapting the game for use at community events such as roadshows. These recommendations emphasise the importance of developing culturally responsive educational tools. In the Singapore context, a culturally responsive game would incorporate features that reflect local values, languages, and experiences of dementia. Examples include the use of familiar local settings (such as hawker centres or HDB estates), inclusion of multilingual options to reflect Singapore’s linguistic diversity, and scenarios featuring intergenerational family interactions, which are a strong feature of Singaporean caregiving culture. Co-design with local caregivers, healthcare professionals, and people living with dementia would also help ensure that the game accurately reflects the nuances of dementia care and community perceptions in Singapore. Such cultural responsiveness is consistent with broader literature emphasising that locally grounded adaptations improve engagement and perceived relevance of dementia awareness programmes [14, 32]. Digital serious games therefore hold potential as flexible and scalable tools to enhance public understanding of dementia across different cultural contexts. By embedding local examples, language options, and collaborative design processes, future practitioners can adapt the Dementia Game model for other communities internationally while maintaining its core educational purpose.

Digital serious games could serve as effective alternatives to conventional methods of disseminating awareness of dementia such as educational lectures. Games are perceived as more enjoyable and have been shown to invoke critical and higher-order learning, leading to a more receptive and active learning of knowledge [13]. In countries where there is a negative stigma or fear associated with dementia [14], the dementia game presents a promising avenue to be a fun and interactive way to change these perceptions.

Evaluating the Dementia Game in Singapore provided valuable insights for cultural adaptation. For instance, participants suggested incorporating multiple language options, reflecting Singapore’s multicultural and multilingual society, as well as adding more structured content to better immerse players in the dementia journey. While the game was effective in challenging stereotypes and promoting a deeper understanding of dementia, the use of interactive scenarios has also been recognised as a useful approach to enhancing dementia awareness in other Asian countries and among children’s nursing students in the UK [29]. Future development of dementia awareness games should also include the perspectives of people living with dementia and their carers in Singapore to ensure relevance and inclusivity.

### Strengths/ limitations

This study employed a qualitative approach to explore participants’ views on dementia before and after playing a game utilising Focus Group Interviews (FGs). The interactive nature of FGs helps probe beliefs and perceptions, and the study benefits from a diverse age and gender participant pool, though most participants are from the Chinese community. Limitations include potential group-think responses and dominant individuals overshadowing others in FGs, although moderators were briefed to mitigate this. The study’s results may lack generalisability due to participants’ specific backgrounds, primarily from Dementia Singapore and allied health and nursing students. Predominantly Chinese ethnicity further narrows applicability of the findings to other ethnic groups. The study’s small sample size of 19, below the intended 20–40 participants, potentially limits the depth and generalisability of findings to Singapore’s wider population. In addition, as the focus group discussions were conducted retrospectively after gameplay, participants’ reflections may have been influenced by recall or hindsight bias, potentially affecting the accuracy or depth of their reported experiences.

## Conclusion

The Dementia Game promoted a greater level of empathetic understanding towards people living with dementia in Singapore. The Game dispelled misconceptions and challenged stereotypes held by the public in Singapore. This study highlights the potential of serious games to address societal challenges and raise dementia awareness in Singapore. However, further research is needed to assess if serious games might lead to behaviour change in supporting persons with dementia to live well in the community.

## Supplementary Information

Supplementary Material 1.

## Data Availability

The datasets used and/or analysed during the current study are available from the corresponding author on reasonable request.
