# Supplementary material for: Exploring the use of a dementia game for raising public awareness in Singapore: a descriptive qualitative study
Source: BMC Public Health. 2026 Feb 16;26:956. doi: 10.1186/s12889-025-26166-7 (PMC13011760; doi:10.1186/s12889-025-26166-7)
Supplement: Supplementary file 1 — Supplementary Material 1. [file 12889_2025_26166_MOESM1_ESM.docx]

**Focus Group Discussion**

Focus group questions

1. Have you had any experience working with people living with dementia?
2. Do you have any relatives, friends or family members with dementia? If there is, what are your experiences interacting or living with them?
3. What was your perception of people living with dementia prior to playing the online game?
4. Did the game change your perception towards people with dementia? In what ways has it changed?
5. With this change in perception, how do you think it will affect your behaviour towards people living with dementia, and your interactions with them?
6. Will you recommend the online game to others?
7. What was your experience like playing the game? Do you have any suggestions to improve the game?
